# Unrelated Hematopoietic Stem Cell Donor Matching Probability and Search Algorithm

**DOI:** 10.1155/2012/695018

**Published:** 2012-11-13

**Authors:** J.-M. Tiercy

**Affiliations:** National Reference Laboratory for Histocompatibility, Transplantation Immunology Unit, Department of Medical Specialties and Department of Genetics and Laboratory Medicine, Geneva University Hospitals, University of Geneva, 1211 Geneva, Switzerland

## Abstract

In transplantation of hematopoietic stem cells (HSCs) from unrelated donors a high HLA compatibility level decreases the risk of acute graft-versus-host disease and mortality. The diversity of the HLA system at the allelic and haplotypic level and the heterogeneity of HLA typing data of the registered donors render the search process a complex task. This paper summarizes our experience with a search algorithm that includes at the start of the search a probability estimate (high/intermediate/low) to identify a HLA-A, B, C, DRB1, DQB1-compatible donor (a 10/10 match). Based on 2002–2011 searches about 30% of patients have a high, 30% an intermediate, and 40% a low probability search. Search success rate and duration are presented and discussed in light of the experience of other centers. Overall a 9-10/10 matched HSC donor can now be identified for 60–80% of patients of European descent. For high probability searches donors can be selected on the basis of DPB1-matching with an estimated success rate of >40%. For low probability searches there is no consensus on which HLA incompatibilities are more permissive, although HLA-DQB1 mismatches are generally considered as acceptable. Models for the discrimination of more detrimental mismatches based on specific amino acid residues rather than specific HLA alleles are presented.

## 1. Introduction

An increasing number of transplantations are now performed with hematopoietic stem cells (HSC) from unrelated volunteer donors. This trend has been largely facilitated by the impressive growth of volunteer donor registries in the last decade: 8 million donors in 2002 and more than 20 million in 2012. The implementation of recipient and donor HLA high resolution genotyping in the clinical practice has clearly contributed to improve the success of transplantation through a better matching [1, 2]. On the other hand the polymorphism of HLA genes turns out to be much higher than anticipated, resulting in larger difficulties in identifying a perfectly matched donor. Because most donors in the Bone Marrow Donor Worldwide (BMDW) registry are of European descent, searches for patients of other ethnic backgrounds have a lower success rate, particularly for those patients with a mixed origin.

HLA matching is commonly based on exons 2 and 3 polymorphism for class I loci and on exon 2 polymorphism for class II loci. The nature of HLA polymorphism with reshuffling of gene segments coding for just a few amino acids has rendered HLA typing a challenging task. The HLA typing techniques currently used in the clinical laboratories often lead to ambiguities because alleles share sequence motifs and because a number of alleles are not resolved by the methods in use. Most typing techniques rely on a locus-specific generic amplification (of one or several exons) which makes it sometimes difficult ot detect whether two polymorphic segments are in *cis* or in *trans* in heterozygous individuals. Furthermore the extension of sequencing techniques to additional exons has disclosed many new alleles, thereby contributing to increase the difficulty of HLA matching. The deleterious impact of single HLA disparities between patient and donor has been largely documented [1–3]. Matching for HLA-A, B, C, DRB1, and DQB1 alleles, a so-called 10/10 match [1–3], and more recently for HLA-DPB1 [2, 4, 5], has been shown to decrease the risk of acute graft-versus-host disease (aGVHD) and mortality after HSCT.

In 2002 we have introduced at the very start of the search an estimation of the probability to identify a perfectly matched donor, that is, compatible for the HLA-A, B, C, DRB1/B3/B5, and DQB1 loci. The probabilities were classified in 3 categories: high (>95% chance), intermediate (about 50%), and low (<5%). As computed from 350 searches (2002–2005) the positive and negative predictive values were 96% and 88%, respectively [6]. This paper reviews our experience in unrelated HSC donor searches as a followup of the search algorithm applied in our laboratory since 2002 [6]. A recent evaluation of the success rate and of the time frame for the identification of a suitable donor as well as the impact of the inclusion of DPB1 matching in the algorithm are presented and compared to those reported by other centers. Criteria that negatively impact the matching probability rate, and HLA-linked parameters that could be taken into account for selecting a mismatched donor, are reviewed. Clinical and functional relevance of HLA disparities is reviewed and possible models for the identification of more detrimental mismatches based on specific amino acid positions are discussed.

## 2. Search Probabilities

According to the search algorithm initiated in 2002 on a national basis, search probabilities are assigned as high, intermediate, or low based on patients HLA-A, B, C, DRB1/B3/B5,DQB1 haplotypes and on interrogation of the BMDW database [6]. Parameters that are taken into account for the probability assignment are presented in the next section. For each consecutive year the relative ratios of high/intermediate/low probabilities have been computed. All donors were requested by the national registry Swiss Blood Stem Cells (SBSC) and tested by the national reference laboratory for histocompatibility (LNRH). Usually 4–6 donors were requested, taking into account a >20% donor unavailability rate.

**Figure 1:**
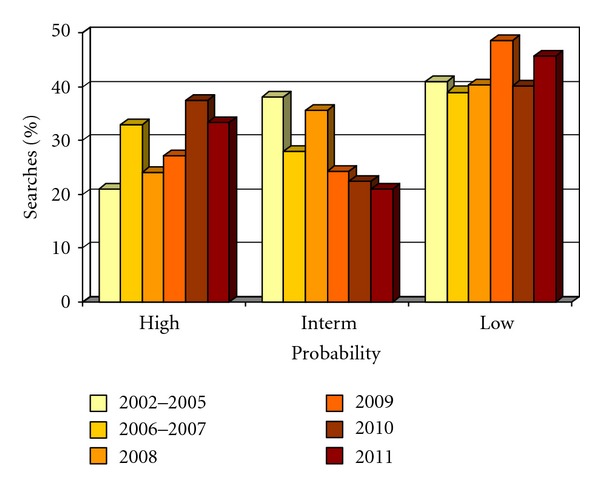
Relative distribution of 1244 high, intermediate, and low probability searches run from 2002 to 2011. The 2002–2005 probability estimates have been reported previously [6].

As compared to the initial observations of 2002–2005, the ratio of high probability searches has increased from 21% to 33–37% in the last 2 years (Figure 1). However the ratio of low probability searches remained stable around 40%. The absolute increase of the registered donors in BMDW, the implementation of HLA typing data (higher resolution level and additional loci tested) of the newly registered donors, and our increased knowledge on HLA haplotypic frequencies [7–12] have also allowed more precise probability estimates. Indeed the ratio of searches qualified as intermediate probability searches (i.e., the most difficult to assign) has decreased from 38% to 21-22% in the last 2 years (Figure 1). Predictive algorithms based on population HLA allele and haplotype frequencies are used by other centers: Haplogic by the National Marrow Donor Program (NMDP), Optimatch by the German Registry or EasyMatch by the French Registry.

## 3. Impact of Rare Alleles and Haplotypes on the Search

Based on our experience of the last 10 years, still 2–5% of the patients do have a unique phenotype (not necessarily including a rare HLA variant) that is not represented in the 20 million donors-BMDW registry. A German study based on 2008-2009 searches reported a 3.3% rate [13]. The ratio is expected to be higher for patients of non-European ancestry. In our experience, among 55 patients with 0 donor in BMDW some serotypes occurred more frequently, such as A25, A33, A68, B18, B53, B58, or B72. HLA-DRB1*09:01-,*10:01-, *14:01, *15:02- and *04:02/03/05/06/07/08-positive haplotypes also occurred more frequenly (data not shown).

Criteria that negatively impact the probability to identify a 10/10 compatible donor are summarized in Table 1 and are obviously linked to patients allele/haplotype frequencies [6, 10, 11, 13]. Searches for patients with a rare allele (e.g., B*07:04 or DRB1*11:58 as encountered in patients analysed in our laboratory) have a low probability of success. Even searches for patients with alleles that represent 5–10% of all alleles within a serotype such as B*35:02 or DRB1*13:03, may have a low probability estimate depending on the extended HLA-A, B, DRB1 haplotype. For example matching for A*02:05 will be much easier if the patient has the A2-B50-DR7 haplotype [11] when compared to the A2-B50-DR3 haplotype. Rare alleles are often associated with a well-defined HLA-A-B-DRB1 haplotype, presumably because of a more recent origin of the allele. A few examples are illustrated in Table 2. A most representative case is the A*02:151 allele, initially described as A*9251 [14], that was subsequently confirmed in 17 individuals (http://www.ebi.ac.uk/imgt/hla/): in 13/17 confirmations this allele was identified on the haplotype A*02:151-B*07:02-C*07:02-DRB1*15:01. Consequently, the presence of a rare allele on a given haplotype might not necessarily mean that search will not be successful. Recently sequenced new alleles that differ outside exons 2 and 3 (for class I) and exon 2 (for class II) may also impact on matching probability. A classic example is the DRB1*14:01 versus *14:54 incompatibility. However the clinical relevance of such disparities is unknown. Unusual B-C and DRB1-DQB1 [6, 11, 13, 15] associations involving common alleles also lead to low probability searches. In such cases the transplant physician should rapidly consider a 9/10 matched donor with a C or DQB1 mismatch, respectively.

## 4. Search Algorithm and DPB1 Matching

An outline of the search algorithm as a function of the probability estimate is represented in Table 3. Requesting >2 donors for the high probability searches has also proven to be useful for the rapid identification of a “back-up donor” since the availability rate of selected donors has slightly decreased in the past years. As a major implementation of our initial algorithm [6], we have recently included HLA-DPB1 typing in the algorithm for a fraction of the high probability searches. Selection according to HLA-DPB1 matching was evaluated on 33 patients for whom >1 potential 10/10 matched donor could be identified (January–July 2012). Based on 33 searches we could identify a DPB1 matched donor for 42.4% of the patients (including one DPB1 mismatched pair in rejection direction only), with an average of 2.7 donors tested/patient (range 1–5, 90 donors tested). Although calculated on a limited number of searches that include essentially patients of European ancestry, this is the first evaluation of the success rate of prospective DPB1 typing aiming at the identification of a 12/12 matched donor. If no DPB1-matched donor can be identified, donors can be selected according the T-cell epitope (TCE)3 matching algorithm [4].

## 5. Efficiency of the Searches

Efficiency of the search is detemined by the likelihood to identify a “matched” donor by testing a “reasonable” number of donors (i.e., in a cost-efficient manner) and by the the time required for the process. Data in the literature on “successful” searches and on search duration are scarce and are difficult to compare mainly because HLA matching criteria vary between the centers. Depending on risk factors such as patient's age, disease stage, or urgency of transplant, a 9/10 matched donor would be considered a suitable donor in center A, but not in center B.

A detailed Dutch study of 212 searches run in 1996–2000 showed that a suitable donor (9-10/10, or <9/10 in 13% cases) could be identified for 69% of the patients with a median search time of 2.5 months [16]. A study from the UK based on 60 unrelated donor searches run in 2005 reported that a 9-10/10 donor could be identified for 72% of the patients with a median time to donor availability of 11 weeks if donor was registered in the UK and of 14 weeks if the donor had to be searched in the international registry [17]. A retrospective evaluation of 549 searches run in 2005 for 23 German transplant centers reported the identification of a 10/10 matched donor for 61.6% of the patients [13]. Overall median search duration was 20 days (7–330), 45 days (7–1225), and 477 (2–2870) days in patients groups with high, low and very low search success probabilities, respectively [13]. A recent Austrian study reported that a 9-10/10 (exceptionally a 8/10) matched donor could be identified for 78.3% of the patients (87.7% of European origin) in 2008–2010 searches, with a mean search time of 1.84 months in 2010 [18].

Not surprisingly ethnic origin of the patients has a major influence on the likelihood to find a matched donor because of the underrepresentation of “non-Caucasian” donors in the international registry. For example, based on the NMDP data, “Asian” patients have a two-fold higher probability to have a mismatched donor compared to “Caucasian” patients [19]. In a single center the donor (7-8/8 match) identification rate was about 90% for patients classified as “US or European Caucasians”, 76% for “Hispanics”, 62% for “Black/African American”, and 33% for “Asians” [20].

In our experience we could identify a 10/10 or 9/10 matched donor in 71.2% patients in 2002–2005 (350 searches, mean 4.9 donors tested/patient) [6], and in 81.8% patients in 2010-2011 (274 searches, mean 5.1 donors tested/patient) (Table 4). In 2011 the average number of tested donors/patient was similar for all 3 categories (4 donors/patient), but lower than in 2010 (data not shown). The efficiency of searches run at the LNRH in 2010-2011 was evaluated by computing the time frame between the start of the search and the date of the HLA report providing the best matched donor, that is, a 10/10 matched donor for the high probability searches, or 9-10/10 matched donor for the low/intermediate searches, and with date of transplantation. For the high probability searches run in 2010-2011 that led to a transplant the average time to propose a donor to the transplant center was 54 days (Table 5). This is comparable to the 1.4 months median search time reported for Northwestern European patients [16] and the 1.7 months time reported for Austrian patients [21]. This duration was however longer than the 21-days mean time reported for successful searches run by the German study [13]. Considering the nontransplanted patients with a high probability estimate the time frame for donor identification was identical. For the intermediate probability searches the time frame was 73 (34–217) days, and for the low probability searches the time frame was 83 (33–308) days (data not shown). For these 2 categories the search time was therefore longer than the average time reported in other studies [13, 21], but comparable to the duration reported by the U.K. study of searches run in 2005 [17]. Interestingly the time to transplant was similar for high and low probability searches, but slightly lower for intermediate probability searches (Table 5).

## 6. Clinical and Functional Relevance of Single HLA Mismatches

Whereas there is a consensus on the negative impact of single mismatches at HLA-A, B, C, DRB1 loci, the most difficult issue in selecting a 9/10 matched donor concerns the nature of the accepted mismatch. HLA-DQB1 incompatibilities are usually more readily accepted [1–3, 22]. In the NMDP study [3] HLA-A and -DRB1 mismatches were reported to have a more detrimental impact on overall survival than HLA-B and -C mismatches. On the other hand a recent analysis of unrelated donor peripheral blood HSC transplants from NMDP reported that only HLA-C antigen and HLA-B allele or antigen mismatches were associated with mortality [23]. In the Japan Marrow Donor Program (JMDP) study, HLA-A/B mismatches, but not HLA-C/DRB1/DQB1, were found to be significantly associated with reduced overall survival [24]. HLA disparities might reveal a stronger negative impact in those patients that have less advanced disease [1–3, 25] or other risk factors. There are no conclusive data showing a difference between allele-level and antigen-level mismatches [3, 26]. Furthermore, one should be careful in interpreting the permissivity of a given locus as identified in retrospective studies, because of possible bias in the accepted mismatches. For example, the role of DRB1 incompatibilities could be underestimated in patients study groups if a significant number of DRB1*11:01 versus *11:04 mismatched pairs are included. It is perhaps not a surprise that the negative impact of HLA-C mismatches is reported with a high statistical significance, as compared to A,B,DRB1 mismatches, since incompatibilities do occur more frequently at HLA-C locus and are often more readily accepted by the transplant centers. A hierarchy in the relevance of HLA incompatibilities must be considered in light of other patient/donor risk factors, as proven by the high predictive value of the EBMT risk score [27, 28]. *A fortiori* the ranking of individual permissive mismatches will be impossible to define unless extremely large patients cohorts can be analysed [29]. Some HLA incompatibilities have been shown to be potential permissive mismatches by *in vitro* cytotoxic T lymphocyte precursor (CTLp) frequency assays, as exemplified by the C*03:03 versus *03:04 disparity [30, 31].

## 7. Evaluation of HLA Mismatches at the Amino Acid Level

Other strategies for disclosing less detrimental mismatches have focused on the nature of the mismatch at the amino acid (aa) level. The HistoCheck scoring system for HLA class I mismatches, based on functional similarity of aa involved in antigenic peptides and T-cell receptor binding turned out not to be predictive of clinical outcome [32]. An evaluation of the impact of individual HLA mismatches, such as those reported in the JMDP study [33] may not be applicable in other populations which show a much larger heterogeneity in HLA disparities and therefore fewer mismatches of similar nature [29]. Using a novel statistical methodology, Marino et al. [34] have reported 13 aa substitutions associated with increased mortality at day 100 in low/intermediate risk patients transplanted with HSC from a single HLA class I mismatched donor. In a recent study [35], the alloreactive CTLp frequency determined in single HLA-A and -C incompatibilities was associated with the aa differences between the mismatched alleles. The probability of a negative CTLp was higher in pairs with >9 aa differences compared to pairs with 0–5 aa differences in the *α*-helices and *β*-sheet. Eight aa (62, 63, 73, 80, 116, 138, 144, 163) were most predictive for a negative CTLp frequency analysis. It is however difficult to compare this model with the random forest analysis mentioned above since 7 of the 12 aa substitutions associated with a negative CTLp outcome are reported to be associated with lower 100 day-survival in the NMDP analysis [34]. At least these models should be tested on independent patients cohorts. CD8+ T-cell alloreactivity, as determined by intracellular staining for IFN-*γ*, has been reported to be higher for HLA-B than for HLA-A mismatches [36]. This observation is not consistent with the more detrimental impact of HLA-A disparities reported in the NMDP study [3].

## 8. Conclusion

As evaluated in searches for patients mainly of European ancestry, a 9-10/10 HLA matched donor can be identified for 60–80% patients. Many transplant centers are now using search algorithms based on allele/haplotype frequencies in order to take earlier decisions to transplant with a mismatched donor or to select an alternative donor (e.g., cord blood, haplo-identical donor) or a nontransplant strategy. In our preliminary experience, the inclusion of prospective HLA-DPB1 typing in the search algorithm for those patients with more than one 10/10 allele matched donor has allowed to identify a 12/12 matched donor for about 40% patients. The challenge remains to reliably predict the functional relevance of individual mismatches for low probability searches, but at least some models are testable. Considering the multiple clinical variables in HSCT, as represented partially by the EBMT risk score [27], it is likely that only clinical studies with more homogenous patients cohorts will be informative. Parameters such as urgency of the transplantation, T-cell depletion, and reduced intensity conditioning might well impact on the role of HLA disparities. At the present time the ranking of HLA-A, B, C, or DRB1 mismatches still appears elusive. We consider the possibility that an *in vitro* functional assay may be used in the algorithm provided it is simple enough, requires limited amount of blood, and is quantitatively highly reproducible. MHC-linked non-HLA genetic polymorphisms that do impact clinical outcome [5, 37, 38] could also be included in the algorithm, primarily for the high probability searches, if validated by larger scale studies.

## Figures and Tables

**Table 1:** Parameters that contribute to define a low probability estimate.

HLA, ethnicity, nb donors	Examples and comments
≤3 donors in BMDW	
Non-European ancestry	
Rare^(1)^ allele at any locus	A*02:17, B*44:05, DRB1*11:03
Rare B-C association	B*18:01-C*02:02, B*51:01-C*16:02
Rare DRB1-DQB1 association	DRB1*15:01-DQB1*06:03, DRB1*0701-DQB1*03:02
B*15:01, B*18:01, B*27:05, B*51:01-positive haplotypes	Higher risk of C MM
B*35:02/35:03/35:08-positive haplotypes	Higher risk of B*35 allele MM

^(1)^<5% of the alleles included a given serotype.

**Table 2:** Examples of conserved haplotypes with rare HLA class I alleles.

Rare allele	First assigned	Extended haplotype
A*02:151	2008	A*02:151-B*07:02-C*07:02-DRB1*15:01
A*03:20	2005	A*03:20-B*51:08-C*16:02-DRB1*11:04
A*03:50	2009	A*03:50-B*35:01-C*04:01-DRB1*01:01
A*03:96	2010	A*03:96-B*07:02-C*07:02-DRB1*15:01
A*03:102	2010	A*03:102-B*18:01-C*02:02-DRB1*13:01
B*07:20	1999	A*24:02-B*07:20-C*07:02-DRB1*16:01
B*27:70	2010	A*02:01-B*27:70-C*02:02-DRB1*04:01/04:04
B*51:43	2006	A*02:01-B*51:43-C*14:02-DRB1*04:01
C*05:14	2006	A*02:01-B*51:01-C*05:14-DRB1*04:04
C*15:13	2004	A*02:01-B*51:01-C*15:13-DRB1*04:02

**Table 3:** Unrelated donor search algorithm for high, intermediate, and low probability categories aiming at the identification of 12/12, 10/10, or 9/10 matched donors. This algorithm is based on requesting blood sample from BMDW registries, and histocompatibility testing in the laboratory serving the transplant center(s). Alternatively HLA typing can be performed by the laboratory linked to each registry at the request of the transplant center. Intermediate resolution typing must resolve the main allele groups, for example, B*44:02 versus B*44:03 groups or C*07:01 versus 07:02 groups.

Probability	Steps	Procedure
High	1	Urgent transplant:
		(i) select 2–4 donors (incl. “back-up donor”) according to age, sex, CMV status, blood group
		(ii) type for HLA-A, B, C, DRB1/B3/B5, DQB1 at a high resolution level^(1)^
	2	Nonurgent transplant: consider DPB1 matching (a 12/12 match is possible for >1/3 patients)
		(i) type for HLA-A, B, C, and DRB1, DQB1 at an intermediate resolution level
		(ii) type for DRB3 if DRB3 MM risk (i.e. DRB1*13:01 haplotypes)^(1)^
		(iii) if DPB1 matched donor found: complete high resolution typing for all HLA loci

Interm	1	Select 4–6 potential donors and type for HLA-A, B, C, DRB1, and DQB1 at an intermediate level
		>1 potentially matched donor identified: select according non HLA criteria and complete high resolution typing
	2	no matched donor identified and urgent transplant: select according non HLA criteria and complete high resolution typing
	3	no matched donor identified and non-urgent transplant: request another set of 4–6 donors

Low	1	Consider a mismatch early in the search and request 4–6 donors:
		(i) type for HLA-A, B, C, DRB1/B3/B5, DQB1 at an intermediate resolution level
	2	No matched donor identified and urgent transplant:
		(i) select a donor among potential donors with single MM and complete high resolution typing
	3	No matched donor identified and nonurgent transplant:
		(i) request another 4–6 donors and type for HLA-A, B, C, DRB1/B3/B5, and DQB1 at an intermediate resolution level
	4	If no potential donors available in BMDW:
		(i) select donor(s) with a mismatch located at the locus where the patient's rare allele is found
		(ii) if B MM: select donors with B MM associated with same HLA-C (e.g., B35:08 versus B*53:01 or B*13:01 versus B*57:01)
		(iii) if DRB1 MM : select donors with DRB1 mismatches associated with same DQB1 allele (e.g., DRB1*11:03 versus DRB1*12:01)
	5	If no mismatch accepted consider another HSC source (cord blood, haplo-identical donor) or a nontransplant protocol

^(1)^HLA-A, B, C, DRB1, and DQB1 testing is performed by PCR-SSO on microbeads arrays (luminex technology, OneLambda HD reagents) by PCR-SSP (Genovision), and by mono-allelic PCR-SBT (Protrans). HLA-DRB3, DRB5, and DPB1 typing is performed by PCR-SSP.

MM : mismatches.

**Table 4:** Donor matching grade for 274 consecutive searches run from 1.1.2010 to 31.8.2012.

Category^(1)^	Nb	Nb donors	Mean nb	10/10	9/10^(2)^	≤8/10 or non
	patients	tested	don/patient			evaluable
High	103	331	3.2	102 (99%)	1 (1%)	0
Interm	61	333	5.45	38 (62.2%)	20 (32.8%)	3 (5%)
Low	110	744	6.76	19 (17.3%)	44 (40%)	47 (42.7%)

Total	274	1408	5.14	159 (58%)	65 (23.7%)	50 (18.3%)

^(1)^For 26 patients classified with a high (*n* = 7), intermediate (*n* = 5), and low (*n* = 14) probability a formal search was not initiated or no donor could be requested or analysed during the same time frame.

^(2)^DRB3 disparities were counted as a mismatch.

**Table 5:** Time frame of donor searches run from 1.1.2010 to 31.8.2012 for transplanted patients with different search probability estimates.

Category	Nb	Time for donor	Time to HSCT	Mean nb donor
	patients	identification (days)	(days)	tested/patient
High^(1)^	66	54 (20–208)	101 (24–428)	4.92
Interm	30	73 (34–217)	76 (11–170)	5.13
Low	36	83 (33–308)	94 (12–298)	5.05

^(1)^For 98/99 high probability searches a 10/10 matched donor could be identified with a mean duration of 56 days (20–208), a transplant date was not (yet) available for 18 patients, 5 patients declined transplantation, 6 patients died, 1 relapsed, 1 was transplanted abroad, 1 was transplanted with a haplo-identical donor.
